# Incidence of Hospitalizations Involving Alcohol Withdrawal Syndrome in a Primary Care Population

**DOI:** 10.1001/jamanetworkopen.2024.38128

**Published:** 2024-10-08

**Authors:** Tessa L. Steel, Theresa E. Matson, Kevin A. Hallgren, Malia Oliver, Helen E. Jack, Douglas Berger, Katharine A. Bradley

**Affiliations:** 1 Department of Medicine, Division of Pulmonary, Critical Care and Sleep Medicine, University of Washington, Seattle; 2 Harborview Medical Center, Seattle, Washington; 3 Kaiser Permanente Washington Health Research Institute, Seattle; 4 Department of Psychiatry and Behavioral Sciences, University of Washington, Seattle; 5 Department of Medicine, Division of General Internal Medicine, University of Washington, Seattle; 6 Veterans Affairs Puget Sound Health Care System, Seattle Division, Seattle, Washington

## Abstract

**Importance:**

Alcohol withdrawal syndrome (AWS) is an important cause and complication of hospitalizations. Although common and preventable, the incidence of AWS during hospitalizations is poorly described.

**Objective:**

To evaluate the incidence and proportional incidence of hospitalizations involving AWS in an adult primary care population overall and across patient characteristics.

**Design, Setting, and Participants:**

This retrospective cohort study used electronic health records and insurance claims from Kaiser Permanente Washington (KPWA) between July 1, 2018, and June 30, 2022. The study included adults with 1 or more primary care visits during this period or the year prior, where primary care included annual standardized alcohol screening using the Alcohol Use Disorders Identification Test–Consumption (AUDIT-C).

**Exposures:**

Age, sex, race, Hispanic ethnicity, AUDIT-C scores, and comorbid diagnoses.

**Main Outcome and Measures:**

Hospitalizations involving AWS were defined by diagnosis codes documented during hospitalizations (incidence numerator). Time enrolled in KPWA determined person-enrolled-years (incidence denominator). Proportional incidence was calculated as the incidence of hospitalizations involving AWS divided by the incidence of all-cause hospitalizations. Proportional incidence was also estimated for hospitalizations involving other common chronic conditions (chronic obstructive pulmonary disease, diabetes, heart failure, and hypertension), which were also defined using hospital diagnosis codes.

**Results:**

Among 544 825 adults engaged in primary care (mean [SD] age, 47.0 [17.9] years; 310 069 [56.9%] female; 3656 [0.7%] American Indian or Alaska Native, 55 206 [10.1%] Asian, 25 406 [4.7%] Black, 5204 [1.0%] Native Hawaiian or Other Pacific Islander, 365 780 [67.1%] White, 19 791 [3.6%] multiracial, 15 963 [2.9%] other races, and 53 819 [9.9%] unknown race; 33 987 [6.2%] Hispanic, 414 269 [76.0%] not Hispanic, and 96 569 [17.7%] unknown ethnicity), incidence of hospitalizations involving AWS was 169 (95% CI, 159-179) per 100 000 person-enrolled-years overall but as high as 15 347 (95% CI, 13 502-17 331) in patients with other alcohol-attributable diagnoses. The proportional incidence of hospitalizations involving AWS was 2.3% overall, with variation by age, sex, and AUDIT-C scores (eg, 9%-11% in male patients aged 30-49 years and 23%-44% in patients with high-risk AUDIT-C scores of 7-12 points). In most cases, among adults younger than 60 years, proportional incidence of hospitalizations involving AWS matched or surpassed that of other common chronic conditions (chronic obstructive pulmonary disease, diabetes, heart failure, and hypertension).

**Conclusions and Relevance:**

In this cohort study of a large primary care population served by an integrated health system, AWS hospitalizations were common, especially in male patients, younger age groups, and individuals with high-risk alcohol use. During hospitalizations, the burden of AWS was similar to or exceeded complications of other chronic diseases that receive greater medical attention.

## Key Points

**Question:**

How often do primary care patients in an integrated health system experience hospitalizations involving alcohol withdrawal syndrome (AWS)?

**Findings:**

In this cohort study of more than 500 000 adult primary care patients, AWS was common during hospitalizations, especially in men aged 30 to 49 years (9%-11% of hospitalizations) and individuals with high-risk alcohol screening (23%-44% of hospitalizations). Alcohol withdrawal syndrome was generally as common or more common than complications of other chronic conditions (eg, diabetes, hypertension) during hospitalizations in patients younger than 60 years.

**Meaning:**

Alcohol withdrawal syndrome is common during hospitalizations, highlighting opportunities for health system interventions to improve prevention and treatment of alcohol use disorder and its consequences.

## Teaser

This cohort study evaluates the incidence of hospitalizations involving alcohol withdrawal syndrome among adult primary care patients.

## Introduction

Alcohol withdrawal syndrome (AWS) is a highly morbid,^1,2^ potentially fatal^3^ cause and complication of hospitalizations. Alcohol withdrawal syndrome is indicative of physical dependence on alcohol, a diagnostic criterion for alcohol use disorder (AUD). Alcohol use disorder is associated with a host of health risks beyond AWS, including organ failure, severe infections, suicide, and death.^1,4,5,6,7,8^ Complications of AUD are increasing, frequently lead to hospitalization,^5,6,9^ and have been linked to declining life expectancy in the US.^10,11,12,13,14^ Although common and preventable, little is known about the incidence of hospitalizations involving AWS, particularly in primary care populations served by health care systems that could support proactive identification and management of AUD and its complications.^15^

Few studies have evaluated the epidemiology of inpatient AWS. A brief report from a multihospital system in Delaware found that rates of inpatient AWS increased by 34% during the COVID-19 pandemic.^16^ Other studies describing prevalence or incidence of inpatient AWS have focused on single hospitals^17,18,19,20^ or hospitalizations within the Veterans Health Administration (VHA).^21,22^ In 2013, the estimated prevalence of inpatient AWS was 5% to 6% in a national sample of VHA patients that was 96% male with a mean age of 68 years. These prior studies have limited generalizability.

For several common chronic conditions (chronic obstructive pulmonary disease [COPD], diabetes, heart failure, and hypertension), prevention of disease-related hospitalizations is both a treatment goal and measure of (poor) quality care that can be monitored by health care systems.^15,23,24,25^ Similar to hospitalizations for acute complications of diabetes or heart failure, inpatient AWS indicates opportunities for improvement of another common chronic disease—AUD. Despite increased focus on identification and treatment of AUD in primary care settings,^26^ however, less attention has been given to prevention of hospitalizations (and rehospitalizations) involving AWS as a treatment outcome or quality measure.

This study primarily evaluated the incidence of hospitalizations involving AWS across age, sex, race, ethnicity, patient-reported alcohol screening scores, and comorbid diagnoses in a large primary care sample served by an integrated health care system. Secondarily, the incidence of hospitalizations involving AWS was compared with the incidence of hospitalizations involving other common chronic conditions conventionally used to monitor the quality of primary care: COPD, diabetes, heart failure, and hypertension.^27^

## Methods

### Study Design and Setting

This was a retrospective cohort study of patients seen in primary care in Kaiser Permanente Washington (KPWA), which is an integrated health system that included 30 primary care clinics across Washington State at the time of this study. Kaiser Permanente Washington serves diverse individuals, with distributions of age, sex, race, and Hispanic ethnicity that are similar to the population of Washington.^28^ As a payer and provider of health care services, KPWA has outpatient electronic health record (EHR) data and both inpatient and outpatient insurance claim data. Between 2015 and 2018, KPWA implemented standardized protocols for annual alcohol screening in primary care using the 3-question Alcohol Use Disorders Identification Test–Consumption (AUDIT-C).^29,30^ Annual AUDIT-C screening rates among primary care patients have since exceeded 80%.^30,31^ The KPWA Health Research Institute’s Institutional Review Board approved the study with a waiver of consent and Health Insurance Portability and Accountability Act authorization due to minimal risk to human participants and impracticability of conducting the research without a waiver. This report follows the Strengthening the Reporting of Observational Studies in Epidemiology (STROBE) reporting guidelines for observational studies.

### Sample

Adults (aged ≥18 years) insured by a KPWA health plan between July 1, 2018, and June 30, 2022, with at least 1 clinician visit in a primary care clinic during the same period or prior year were included. The sample was restricted to primary care patients to evaluate the association between AUDIT-C scores and hospitalizations involving AWS and based on guidelines recommending prevention and treatment of AUD in primary care settings.^26,32,33,34^ Individuals were excluded if their hospitalization records were not captured by KPWA claims data (ie, 7% of patients covered by managed Medicaid plans).

### Measures

#### Primary Outcome: AWS Hospitalizations

For the main analyses, AWS hospitalizations were defined by primary or secondary AWS billing diagnosis codes (ie, *International Statistical Classification of Diseases and Related Health Problems, Tenth Revision* [*ICD-10*] codes) (eTable 1 in Supplement 1), documented any time during a hospitalization from July 1, 2018, to June 30, 2022. Only hospitalizations 24 hours or longer were included, and individuals could contribute more than 1 AWS hospitalization.^35,36^ We used primary or secondary diagnosis codes to define the primary outcome because AWS is often considered a secondary complication during hospitalizations focused primarily on other medical or surgical issues.^20^

#### Secondary Outcomes: Hospitalizations Involving ACSCs

To compare hospitalizations involving AWS with those involving other common chronic conditions, we used published *ICD-10* codes to identify ambulatory care sensitive conditions (ACSCs): COPD, diabetes, heart failure, and hypertension (eTable 1 in Supplement 1).^27^ Because hospitalizations for ACSCs are conventionally defined using only primary hospital diagnosis codes,^27^ for secondary analyses comparing inpatient AWS and ACSCs, hospitalizations for AWS were likewise defined by primary diagnosis codes only. This approach was chosen to facilitate comparisons between AWS and ACSCs, acknowledging the likelihood that clinically significant AWS would be disproportionately underestimated; AWS coded as secondary likely reflects a true complication during hospitalization, whereas diabetes or heart failure (for example) coded as secondary may reflect a complication or merely ongoing management of a stable comorbidity.

#### Other Measures

Time enrolled in a KPWA health plan during the study was used to compute person-enrolled-years. Demographic characteristics collected from patients and documented in the EHR by the health system were used to approximate patients’ identities and lived experiences. These characteristics included age at hospital admission (18-29, 30-39, 40-49, 50-59, 60-69, and ≥70 years), sex listed on forms of identification used for health plan enrollment (female or male), self-identified race (American Indian or Alaska Native, Asian, Black, Native Hawaiian or Other Pacific Islander, White, multiracial [ie, multiple race selections], other racial groups [cannot be further specified], or unknown), and Hispanic or non-Hispanic ethnicity. The AUDIT-C scores (0-12 points) are the sum of scores for 3 questions about frequency and quantity of alcohol use (0-4 points each). Patients were categorized as no AUDIT-C completed, no alcohol use (0 points), or low (1-2 points for women and 1-3 points for men), unhealthy (3-6 points for women and 4-6 points for men), high-risk (7-8 points), or very high-risk (9-12 points) use.^7,37,38^ For patients with more than 1 AUDIT-C screen during the study period, the first screen was selected. Outpatient *ICD-10* codes documented any time during the study period were used to identify common comorbid diagnoses associated with AUD (eTable 1 in Supplement 1).^39,40,41^ All-cause hospitalizations were defined in the same manner as hospitalizations involving AWS or ACSCs but without restriction to specific diagnoses (eTable 2 in Supplement 1).

### Statistical Analysis

To describe and evaluate patterns of hospitalizations involving AWS, we estimated the incidence in the overall sample and across age categories. Incidence rates were calculated by dividing the total number of events (ie, eligible hospitalizations) by person-enrolled-years and multiplying by 100 000 to express events per 100 000 person-enrolled-years. Proportional incidence was calculated by dividing the incidence of AWS by the incidence of all-cause hospitalizations. The CIs were estimated using nonparametric bootstrapping with 10 000 resamples. We analyzed the incidence and proportional incidence of hospitalizations involving AWS across demographic and clinical characteristics. Furthermore, we stratified age-specific proportional incidence estimates by sex, race, ethnicity, and patient-reported levels of alcohol consumption (AUDIT-C categories) to understand patterns within intersections of these factors. Finally, we estimated incidence and proportional incidence of ACSCs for comparison to AWS estimates. As above, because ACSCs are conventionally measured using only primary diagnoses, hospitalizations involving AWS vs ACSCs were secondarily defined using only primary diagnosis codes. All analyses were conducted using R, version 4.2.3 (R Project for Statistical Computing).

## Results

Characteristics of the KPWA sample (N = 544 825) are presented in Table 1. Of the included adults, 280 811 (51.5%) were enrolled in KPWA care for more than 3 years. Sample eligibility required at least 1 primary care visit; 453 589 patients (83.3%) had at least 2 primary care encounters with the health care system during the study period (median [IQR], 5 [2-11] encounters). The sample spanned all age groups, with the largest being aged 18 to 29 years (114 900 [21.1%]) and smallest being 70 years or older (63 402 [11.6%]); the mean (SD) age was 47.0 (17.9) years. A total of 310 069 patients (56.9%) were female, 234 754 (43.1%) male, and 2 (<0.01%) of unknown sex; 3656 (0.7%) identified as American Indian or Alaska Native, 55 206 (10.1%) Asian, 25 406 (4.7%) Black, 5204 (1.0%) Native Hawaiian or Other Pacific Islander, 365 780 (67.1%) White, 19 791 (3.6%) multiracial, 15 963 (2.9%) other races (cannot be further specified), and 53 819 (9.9%) unknown race; 33 987 (6.2%) were Hispanic, 414 269 (76.0%) non-Hispanic, and 96 569 (17.7%) of unknown ethnicity. Anxiety (157 370 [28.9%]) and mood (115 314 [21.2%]) disorders were the most common outpatient diagnoses evaluated, followed by chronic pulmonary conditions (75 240 [13.8%]), diabetes (57 621 [10.6%]), and cancer (36 338 [6.7%]). Only 15 333 patients (2.8%) had a documented AUD diagnosis, but 118 915 (21.8%) screened positive for unhealthy or greater alcohol use (AUDIT-C score >2 points for women and >3 points for men).

**Table 1.** Characteristics of the Study Patients

Characteristic	No. (%) of patients (N = 544 825)
Age group, y	
18-29	114 900 (21.1)
30-39	100 175 (18.4)
40-49	85 056 (15.6)
50-59	92 925 (17.1)
60-69	88 367 (16.2)
≥70	63 402 (11.6)
Sex	
Female	310 069 (56.9)
Male	234 754 (43.1)
Unknown	2 (<0.01)
Race	
American Indian or Alaska Native	3656 (0.7)
Asian	55 206 (10.1)
Black	25 406 (4.7)
Native Hawaiian or Other Pacific Islander	5204 (1.0)
White	365 780 (67.1)
Multiracial	19 791 (3.6)
Other^a^	15 963 (2.9)
Unknown	53 819 (9.9)
Ethnicity	
Hispanic	33 987 (6.2)
Not Hispanic	414 269 (76.0)
Unknown	96 569 (17.7)
Time enrolled in KPWA during the study period, y	
>3	280 811 (51.5)
1-3	172 027 (31.6)
<1	91 987 (16.9)
Outpatient diagnoses	
Alcohol-attributable diagnoses^b^	1749 (0.3)
Alcohol use disorder	15 333 (2.8)
Anxiety disorder	157 370 (28.9)
Cancer (other than nonmelanoma skin cancer)	36 338 (6.7)
Cerebrovascular disease	19 225 (3.5)
Chronic kidney disease	30 083 (5.5)
Chronic liver disease	28 066 (5.2)
Chronic pulmonary conditions	75 240 (13.8)
Dementia	10 436 (1.9)
Diabetes	57 621 (10.6)
Heart failure	19 049 (3.5)
Mood disorder (depression, bipolar)	115 314 (21.2)
Nicotine use disorder	35 530 (6.5)
Other substance use disorder	11 535 (2.1)
Peptic ulcer disease	3964 (0.7)
Posttraumatic stress disorder	16 210 (3.0)
Psychotic disorder	3357 (0.6)
Alcohol consumption (outpatient AUDIT-C screening scores, 0-12 points)	
Very high-risk use (9-12 points)	3740 (0.7)
High-risk use (7-8 points)	8828 (1.6)
Unhealthy use (3-6 points female/4-6 points male)	118 915 (21.8)
Low use (1-2 points female/1-3 points male)	195 127 (35.8)
No use (0 points)	128 518 (23.6)
No AUDIT-C screen completed	89 697 (16.5)

Abbreviations: AUDIT-C, Alcohol Use Disorders Identification Test–Consumption; KPWA, Kaiser Permanente Washington.

^a^ Other races cannot be further specified.

^b^ Alcohol-attributable diagnoses include alcoholic psychosis, alcohol abuse, alcohol dependence syndrome, alcohol polyneuropathy, degeneration of the nervous system due to alcohol use, alcoholic myopathy, alcohol cardiomyopathy, alcoholic gastritis, alcoholic liver disease, alcohol-induced pancreatitis, and fetal alcohol syndrome (eTable 1 in Supplement 1).^41^

The incidence (reported per 100 000 person-enrolled years hereafter) of all-cause hospitalizations in the sample was 7306 (95% CI, 7239-7376) (eTable 2 in Supplement 1), with 108 455 all-cause hospitalizations during the study period. The overall incidence of hospitalizations involving AWS was 169 (95% CI, 159-179) (Table 2), with 1639 adults experiencing inpatient AWS, of whom 437 (26.7%) had more than 1 hospitalization involving AWS during the study period. The proportional incidence of hospitalizations involving AWS among all-cause hospitalizations was 2.3% (95% CI, 2.2%-2.4%).

**Table 2.** Incidence of Hospitalizations Involving AWS, Overall and by Patient Characteristics

Characteristic	IR (95% CI) for AWS hospitalizations per 100 000 person-enrolled years	PI (95% CI) of AWS relative to all-cause hospitalizations, %^a^
Total	168.6 (158.5-179.2)	2.3 (2.2-2.4)
Age group, y		
18-29	88.9 (73.6-105.7)	2.1 (1.7-2.5)
30-39	167.6 (143.4-193.2)	3.2 (2.7-3.6)
40-49	229.5 (197.1-263.8)	6.7 (5.8-7.7)
50-59	231.9 (203.7-262.5)	4.7 (4.1-5.2)
60-69	178.6 (155.1-203.5)	2.2 (1.9-2.5)
≥70	104.0 (84.3-125.4)	0.5 (0.4-0.6)
Sex		
Female	116.6 (104.6-129.0)	1.5 (1.4-1.7)
Male	236.6 (218.3-254.9)	3.5 (3.2-3.7)
Race		
American Indian or Alaska Native	218.4 (98.5-362.5)	2.4 (1.1-3.9)
Asian	22.4 (12.2-35.3)	0.5 (0.3-0.8)
Black	124.0 (88.2-164.8)	1.5 (1.1-2.0)
Native Hawaiian or Other Pacific Islander	14.7 (0.0-36.9)	0.2 (0.0-0.5)
White	196.9 (183.3-210.6)	2.5 (2.3-2.6)
Multiracial	116.8 (74.6-163.6)	1.9 (1.2-2.6)
Other races^b^	81.5 (51.7-115.4)	1.5 (1.0-2.1)
Unknown	197.9 (159.8-239.4)	3.7 (3.0-4.4)
Ethnicity		
Hispanic	151.1 (113.0-193.1)	2.4 (1.8-3.1)
Not Hispanic	166.2 (154.7-177.7)	2.1 (2.0-2.3)
Unknown	192.9 (163.5-225.4)	4.0 (3.4-4.7)
Outpatient diagnoses		
Alcohol-attributable diagnoses^c^	15 346.7 (13 502.2-17 331.4)	39.3 (35.8-42.9)
Alcohol use disorder	5088.9 (4763.1-5419.8)	25.5 (24.1-26.9)
Anxiety disorder	398.3 (367.9-429.8)	4.1 (3.8-4.4)
Cancer (other than nonmelanoma skin cancer)	188.0 (146.3-235.6)	0.9 (0.7-1.1)
Cerebrovascular disease	421.4 (337.8-514.6)	1.3 (1.0-1.5)
Chronic kidney disease	263.2 (208.4-325.3)	0.9 (0.7-1.1)
Chronic liver disease	1226.9 (1102.8-1361.8)	6.2 (5.6-6.8)
Chronic pulmonary conditions	282.5 (249.1-317.0)	1.9 (1.7-2.2)
Dementia	536.4 (401.8-689.7)	1.2 (0.9-1.6)
Diabetes	228.9 (192.7-267.9)	1.3 (1.1-1.5)
Heart failure	525.3 (433.4-626.6)	1.1 (0.9-1.3)
Mood disorder (depression, bipolar)	508.5 (467.5-551.4)	4.1 (3.8-4.4)
Nicotine use disorder	1051.3 (953.5-1155.3)	7.2 (6.5-7.9)
Other substance use disorder	2385.1 (2101.4-2682.0)	9.1 (8.0-10.1)
Peptic ulcer disease	937.3 (692.0-1200.9)	2.5 (1.9-3.3)
Posttraumatic stress disorder	853.4 (702.7-1014.8)	6.9 (5.7-8.1)
Psychotic disorder	1256.2 (914.9-1639.3)	3.8 (2.8-4.9)
Alcohol consumption (outpatient AUDIT-C screening scores, 0-12 points)		
Very high-risk alcohol use (outpatient AUDIT-C screening scores, 9-12 points)	5754.5 (5023.4-6543.8)	43.5 (39.5-47.6)
High-risk alcohol use (outpatient AUDIT-C screening scores, 7-8 points)	1241.7 (1036.6-1466.1)	22.5 (19.2-26.0)
Unhealthy alcohol use (outpatient AUDIT-C screening scores, 3-6 points for women and 4-6 points for men)	210.3 (187.1-235.1)	4.2 (3.7-4.6)
Low alcohol use (outpatient AUDIT-C screening scores, 1-2 points for women and 1-3 points for men)	59.0 (49.9-68.7)	0.9 (0.8-1.1)
No alcohol use (outpatient AUDIT-C screening scores, 0 points)	91.4 (75.7-108.8)	0.8 (0.7-1.0)
No AUDIT-C screen completed	152.8 (127.2-180.0)	2.3 (1.9-2.7)

Abbreviations: AUDIT-C, Alcohol Use Disorders Identification Test-Consumption; AWS, alcohol withdrawal syndrome; IR, incidence rate; PI, proportional incidence.

^a^ Proportional incidence of AWS: incidence of AWS hospitalizations divided by incidence of all-cause hospitalizations; all-cause hospitalization data in eTable 2 in Supplement 1.

^b^ Other races cannot be further specified.

^c^ Alcohol-attributable diagnoses: alcoholic psychosis, alcohol abuse, alcohol dependence syndrome, alcohol polyneuropathy, degeneration of the nervous system due to alcohol use, alcoholic myopathy, alcohol cardiomyopathy, alcoholic gastritis, alcoholic liver disease, alcohol-induced pancreatitis, fetal alcohol syndrome (eTable1 in Supplement 1).^41^

### Incidence of AWS Hospitalizations

Rates of hospitalizations involving AWS varied by age, sex, race, and AUDIT-C scores (Table 2). Male patients experienced higher incidence of hospitalizations with AWS vs female patients (237 [95% CI, 204-263] vs 117 [95% CI, 105-129]). Individuals identifying as American Indian or Alaska Native or White had higher incidence of hospitalizations involving AWS (incidence rates [IRs], reported per 100 000 person-enrolled years hereafter, 218.4 [95% CI, 98.5-362.5] and 196.9 [95% CI, 183.3-210.6], respectively) compared with those identifying as Asian or Native Hawaiian or Other Pacific Islander (IRs, 22.4 [95% CI, 12.2-35.3] and 14.7 [95% CI, 0.0-36.9], respectively). The incidence of AWS hospitalizations among individuals identifying as Black, multiracial, or other races fell between the aforementioned groups (IRs, 124.0 [95% CI, 88.2-164.8], 116.8 [95% CI, 74.6-163.6], and 81.5 [95% CI, 51.7-115.4], respectively). There was not strong evidence to suggest variation by Hispanic ethnicity. High incidence of hospitalizations with AWS occurred in patients with other alcohol-attributable diagnoses (IR, 15 346.7 [95% CI, 13 502.2-17 331.4]) and/or diagnosed AUD (IR, 5088.9 [95% CI, 4763.1-5419.8]). Other outpatient diagnoses associated with high incidence of hospitalizations involving AWS included other substance use disorders, chronic liver disease, psychotic disorders, and nicotine use disorders. Although individuals reporting no or low alcohol use had relatively low incidence of hospitalizations involving AWS (IR, 91.4 [95% CI, 75.7-108.8] and 59.0 [95% CI, 49.9-68.7], respectively), the incidence was progressively higher as AUDIT-C scores increased from unhealthy (3-6 points for women and 4-6 points for men) (IR, 210.3 [95% CI, 187.1-235.1]) to high-risk (7-8 points) (IR, 1241.7 [95% CI, 1036.6-1466.1]) to very high-risk (9-12 points) (IR, 5754.5 [95% CI, 5023.4-6543.8]) alcohol use.

### Proportional Incidence of AWS Among All Hospitalizations

**Figure 1.**
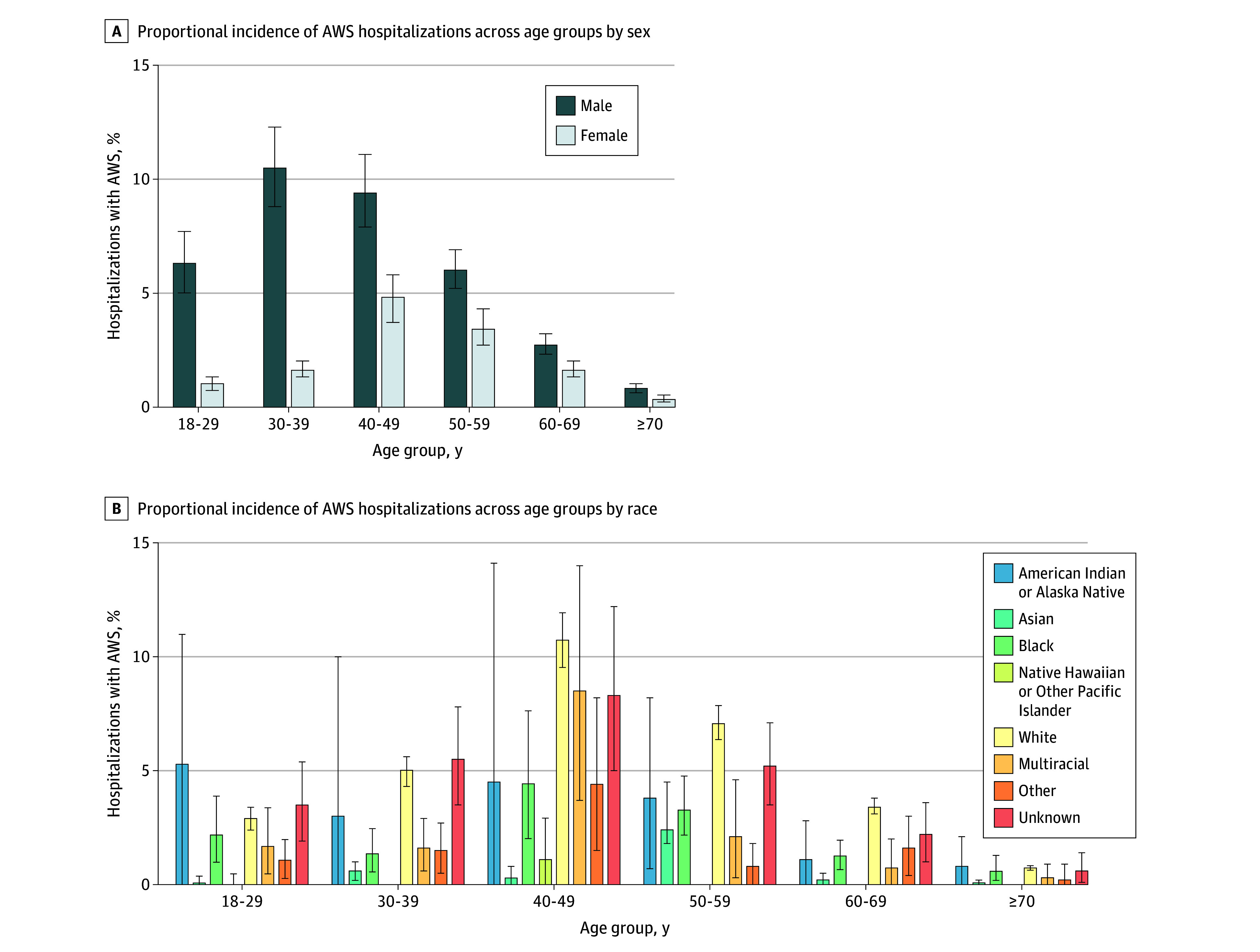
Variation in the Proportional Incidence of Hospitalizations Involving Alcohol Withdrawal Syndrome (AWS) Across Age Groups by Sex and Race Proportional incidence is relative to all-cause hospitalizations. Alcohol withdrawal syndrome is defined by primary or secondary hospital diagnosis codes. Error bars indicate 95% CIs. Precise estimates and CIs are given in eTables 3 and 4 in Supplement 1.

**Figure 2.**
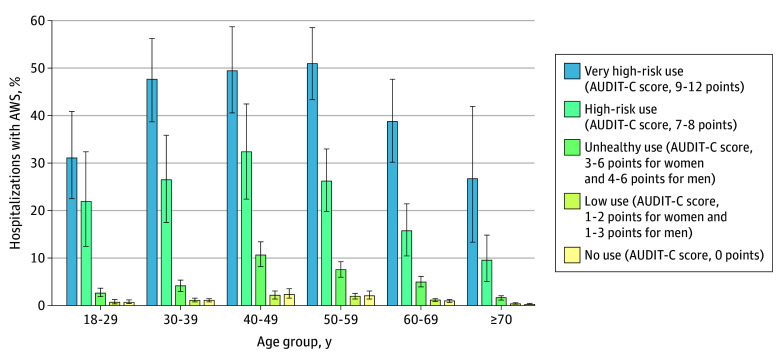
Proportional Incidence of Hospitalizations Involving Alcohol Withdrawal Syndrome (AWS) Across Age Groups by Alcohol Use Disorders Identification Test–Consumption (AUDIT-C) Screening Scores Proportional incidence is relative to all-cause hospitalizations. Alcohol withdrawal syndrome is defined by primary or secondary hospital diagnosis codes. Error bars indicate 95% CIs. Precise estimates and CIs are given in eTable 5 in Supplement 1.

The proportional incidence of hospitalizations involving AWS varied across age groups, by sex, and by race (Figure 1; eTables 3 and 4 in Supplement 1). Proportional incidence of hospitalizations involving AWS was highest at midlife (ages 40-49 years) and lowest with advanced age (ages ≥70 years). These findings held across racial groups. Male patients aged 18 to 39 years experienced more than 6 times the proportional incidence of hospitalizations involving AWS as comparable female patients and roughly double the proportional incidence in other age groups (Figure 1A; eTable 3 in Supplement 1). Peak proportional incidence of hospitalizations involving AWS also occurred earlier in male patients (ages 30-39 years in men vs 40-49 years in women). Notably, 9.4% (95% CI, 7.9%-11.1%) to 10.5% (8.8%-12.3%) of hospitalizations in male patients aged 30 to 49 years involved AWS. The proportional incidence of hospitalizations involving AWS was highest in patients with alcohol-attributable diagnoses (39.3%; 95% CI, 35.8%-42.9%), diagnosed AUD (25.5%; 95% CI, 24.1%-26.9%), and AUDIT-C scores in the high-risk (22.5%; 95% CI, 19.2%-26.0%) and very high-risk (43.5%; 95% CI, 39.5%-47.6%) categories, across all age groups but especially in patients aged 30 to 59 years (Table 2 and Figure 2; eTable 5 in Supplement 1).

### Comparing Proportional Incidence of AWS With Other Common Chronic Conditions

**Figure 3.**
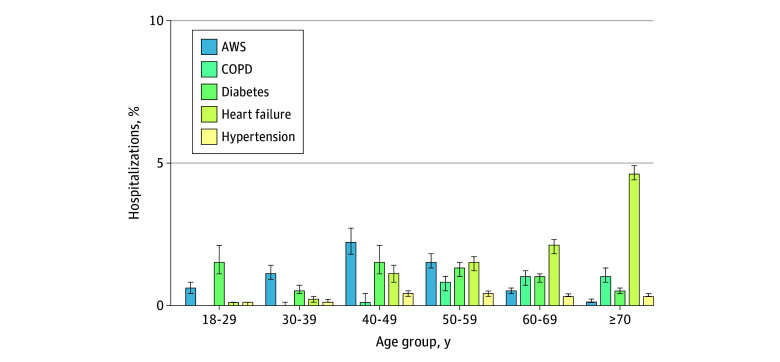
Proportional Incidence of Hospitalizations Due to Alcohol Withdrawal Syndrome (AWS), Chronic Obstructive Pulmonary Disease (COPD), Diabetes, Heart Failure, and Hypertension Across Age Groups Proportional incidence is relative to all-cause hospitalizations. Each outcome is defined using primary diagnosis codes only (consistent with the conventional approach used for measuring ambulatory care sensitive conditions^27^). Error bars indicate 95% CIs. Precise estimates and CIs are given in eTable 6 in Supplement 1.

Overall, the proportional incidence of hospitalizations attributed to AWS vs ACSCs were similar (using primary diagnosis codes to define each outcome): 0.7% (95% CI, 0.6%-0.8%) for AWS, 0.7% (95% CI, 0.6%-0.8%) for COPD, 0.9% (95% CI, 0.8%-1.0%) for diabetes, 2.5% (95% CI, 2.3%-2.6%) for heart failure, and 0.3% (95% CI, 0.3%-0.3%) for hypertension (eTable 6 in Supplement 1); however, these estimates varied substantially by age (Figure 3). Alcohol withdrawal syndrome was as common or more common than most ACSCs during hospitalizations of patients younger than 60 years.

## Discussion

This study examined the incidence of hospitalizations involving AWS in a large, diverse primary care population in an integrated health care system. Approximately 2% of adult hospitalizations involved AWS, with substantial variation by age, sex, race, and outpatient alcohol screening scores. In male patients aged 30 to 49 years, approximately 9% to 11% of hospitalizations involved AWS. Among more than 12 000 individuals with high-risk or very high-risk alcohol screening scores, approximately 23% to 44% of hospitalizations involved AWS. In most cases, among adults younger than 60 years, rates of hospitalization for AWS matched or surpassed rates of hospitalization for complications of other common chronic conditions (ie, COPD, diabetes, heart failure, and hypertension).

This study’s findings markedly augment the scant existing literature describing the epidemiology of inpatient AWS. In prior studies of VHA hospitalizations, involving predominantly male veterans, the overall prevalence of inpatient AWS was approximately 6%, with highest risk occurring at ages 40 to 49 years^21,22^ rather than ages 30 to 39 years in male patients served by KPWA. Geography may help explain these differences: higher rates of heavy drinking and AUD have been reported in Western regions, where the current study is situated.^21,42^ These differences could also reflect alcohol-related risks and cohort effects specific to veterans and the VHA (eg, specialized mental health resources reaching younger age groups in the VHA system).

Consistent with prior research in the VHA,^22^ patients with alcohol-related diagnoses (including those that are alcohol attributable, such as alcohol cardiomyopathy)^43^ and diagnosed AUD had a high burden of hospitalizations involving AWS (proportional incidence, 39.3% and 25.5%, respectively). In addition, the proportional incidence of hospitalizations involving AWS was elevated in individuals with high-risk and very high-risk AUDIT-C scores (7-8 and 9-12 points: 22.5% and 43.5%, respectively). This finding aligns with existing literature on associations between AUDIT-C alcohol screening scores and hospitalizations for all causes,^7,44,45^ alcohol-related diagnoses,^7^ and other diagnoses.^46,47,48^ This body of research shows that sharp increases in complications of alcohol use are associated with high-risk and very high-risk AUDIT-C scores (7-8 and 9-12 points, respectively),^49,50,51^ consistent with sharply increased rates of AWS identified in these groups in the current study.

In addition to characterizing age- and sex-based distributions of hospitalizations involving AWS, this study identified variation by self-identified race. Reasons for racial disparities involving complications of alcohol use are complex and tied to historical patterns of racism and persistent structural racism, which also vary by geographic location.^52^ The incidence of hospitalizations involving AWS was relatively higher among individuals identifying as American Indian or Alaska Native or White and relatively lower among individuals identifying as Asian or Native Hawaiian or Other Pacific Islander; this finding is consistent with prior literature suggesting higher and lower prevalence of AUD in these racial groups, respectively.^52^ Although these findings add to sparse existing data on race and AWS, small sample sizes after stratification by race and age limited precision of the estimates. Ultimately, this topic requires more nuanced research using larger, targeted samples; race-conscious frameworks; and measures that explicitly address racial discrimination at individual and environmental levels.^53^

Relative to disease burden,^11,54^ AUD has been neglected by the medical profession and health care systems: less than a quarter of patients with AUD receive evidenced-based treatments for their disease.^55,56,57,58,59^ The current study has several implications for clinicians, health system leaders, and policymakers seeking to address these gaps in care. We compared inpatient AWS with hospitalizations for other chronic diseases to assess the magnitude of the problem; this comparison may also suggest appropriate solutions. Hospitalizations for diabetes or heart failure, for example, represent severe manifestations of chronic illnesses that may or may not be previously diagnosed. Hospitalizations often prompt targeted interventions for affected patients, including specific preventive programs for those at highest risk for recurrent complications and health care system–wide efforts to improve disease management (eg, accurate diagnosis, appropriate pharmacotherapy, and behavioral interventions).^15,60,61,62,63^

Similar responses to AWS could improve outcomes of AUD. Outpatient clinicians can identify AUD using the AUDIT-C and an Alcohol Symptom Checklist^34,37,64^ and offer treatment to patients at risk for complications,^30^ including medications, counseling, residential programs, and peer support.^33,65,66,67,68,69^ Inpatient clinicians can anticipate AWS when patients with high-risk alcohol use are admitted to the hospital, consider prophylactic treatment,^70,71^ and use the hospitalization to engage, treat, and support patients in effective transitions to long-term AUD care^8^; these efforts are particularly important for reducing readmissions involving AWS, which are common.^36,68^ Preoperative abstinence programs are effective for improving surgical outcomes,^72,73^ but complementary strategies (eg, reduction in preoperative alcohol use and prophylactic treatment of AWS during unplanned admissions) should also be evaluated. Finally, researchers and health care system leaders could monitor AWS during hospitalizations as a marker of quality care for AUD. Efforts to prevent hospitalizations involving complications of other chronic conditions have led to defined ACSCs,^27^ which are monitored over time to evaluate the accessibility and quality of outpatient care.^15,24,25^ Hospitalizations involving AWS could be similarly used to assess and monitor the population burden of AUD in addition to other indicators of system performance (eg, rates of completed and positive alcohol screening, pharmacotherapy, and counseling).

### Limitations

This study has some limitations. *ICD-10* codes likely underestimate true AWS incidence because AWS is often not the primary focus of hospitalizations and thus is often undocumented or undetected.^20,74,75^ Identification of AWS using *ICD-10* codes may also be biased by stereotypes (eg, unrecognized AWS associated with advanced age, female sex, and/or certain socioeconomic and racial groups).^76^ Unfortunately, no viable alternative methods for identifying AWS in large administrative datasets currently exist. The AUDIT-C scores used in this study could be biased by factors such as social desirability.^77^ Although the reliability of AUDIT-C scores is high in this study’s setting,^78^ patients can change or underreport alcohol use: 2% of hospitalizations of middle-aged adults in this sample who reported no or minimal alcohol use on the AUDIT-C nonetheless involved AWS. This finding could also reflect the timing of AUDIT-C screening, given that the first available AUDIT-C score was used during the 3-year study period. Additionally, this study focused on a single health care system serving an insured population in Washington State, which may limit generalizability given that AUD and AWS vary by geographic, social, economic, and cultural factors.^21,52,79^ Analogous studies in other health care systems are needed.

## Conclusions

In this cohort study of a large primary care population in an integrated health care system, hospitalizations involving AWS were common, particularly in male patients, younger age groups, and individuals with high-risk alcohol screening and/or alcohol-related diagnoses. The incidence and proportional incidence of hospitalizations involving AWS varied considerably by age, sex, race, and outpatient alcohol screening scores. In adults younger than 60 years, the burden of hospitalizations attributed to AWS generally matched or surpassed hospitalizations for other chronic conditions that receive greater medical attention. The high incidence of hospitalizations involving AWS reinforces the need for health care systems to prioritize identification, treatment, and monitoring of AUD.

## Notes

Supplement 1. **eTable 1.** ICD-10 Codes used to define alcohol withdrawal syndrome (AWS), chronic ambulatory care sensitive conditions (ACSCs), and common outpatient diagnoses associated with alcohol use disorder (AUD) **eTable 2.** Incidence of alcohol withdrawal syndrome (AWS) hospitalizations, overall and by patient characteristics, including all-cause hospitalization data **eTable 3.** Proportional incidence of alcohol withdrawal syndrome (AWS) hospitalizations relative to all-cause hospitalizations across age groups by sex **eTable 4.** Proportional incidence of alcohol withdrawal syndrome (AWS) hospitalizations relative to all-cause hospitalizations across age groups by race **eTable 5.** Proportional incidence of alcohol withdrawal syndrome (AWS) hospitalizations relative to all-cause hospitalizations across age groups by Alcohol Use Disorder Identification Test-Consumption (AUDIT-C) category **eTable 6.** Proportional incidence of primary ICD-10 diagnosis codes only for alcohol withdrawal syndrome (AWS), heart failure, chronic obstructive pulmonary disease (COPD), diabetes, and hypertension hospitalizations relative to all-cause hospitalizations across age, race, and ethnicity categories

Supplement 2.
Data Sharing Statement
