# Supplementary material for: Incidence of Hospitalizations Involving Alcohol Withdrawal Syndrome in a Primary Care Population
Source: JAMA Netw Open. 2024 Oct 8;7(10):e2438128. doi: 10.1001/jamanetworkopen.2024.38128 (PMC11581492; doi:10.1001/jamanetworkopen.2024.38128)
Supplement: Supplement 1. — eTable 1. ICD-10 Codes used to define alcohol withdrawal syndrome (AWS), chronic ambulatory care sensitive conditions (ACSCs), and common outpatient diagnoses associated with alcohol use disorder (AUD) eTable 2. Incidence of alcohol withdrawal syndrome (AWS) hospitalizations, overall and by patient characteristics, including all-cause hospitalization data eTable 3. Proportional incidence of alcohol withdrawal syndrome (AWS) hospitalizations relative to all-cause hospitalizations across age groups by sex eTable 4. Proportional incidence of alcohol withdrawal syndrome (AWS) hospitalizations relative to all-cause hospitalizations across age groups by race eTable 5. Proportional incidence of alcohol withdrawal syndrome (AWS) hospitalizations relative to all-cause hospitalizations across age groups by Alcohol Use Disorder Identification Test-Consumption (AUDIT-C) category eTable 6. Proportional incidence of primary ICD-10 diagnosis codes only for alcohol withdrawal syndrome (AWS), heart failure, chronic obstructive pulmonary disease (COPD), diabetes, and hypertension hospitalizations relative to all-cause hospitalizations across age, race, and ethnicity categories [file jamanetwopen-e2438128-s001.pdf]

Supplemental Online Content

Steel TL, Matson TE, Hallgren KA, et al. Incidence of hospitalizations involving alcohol withdrawal syndrome in a primary care population. *JAMA Netw Open*. 2024;7(10):e2438128. doi:10.1001/jamanetworkopen.2024.38128

eTable 1. ICD-10 Codes used to define alcohol withdrawal syndrome (AWS), chronic ambulatory care sensitive conditions (ACSCs), and common outpatient diagnoses associated with alcohol use disorder (AUD)

eTable 2. Incidence of alcohol withdrawal syndrome (AWS) hospitalizations, overall and by patient characteristics, including all-cause hospitalization data

eTable 3. Proportional Incidence of alcohol withdrawal syndrome (AWS) hospitalizations relative to all-cause hospitalizations across age groups, by sex

eTable 4. Proportional Incidence of alcohol withdrawal syndrome (AWS) hospitalizations relative to all-cause hospitalizations across age groups, by race

eTable 5. Proportional Incidence of alcohol withdrawal syndrome (AWS) hospitalizations relative to all-cause hospitalizations across age groups, by Alcohol Use Disorder Identification Test-Consumption (AUDIT-C) category

eTable 6. Proportional Incidence of Primary ICD-10 diagnosis codes only for alcohol withdrawal syndrome (AWS), heart failure, chronic obstructive pulmonary disease (COPD), diabetes, and hypertension hospitalizations relative to all-cause hospitalizations across age, race, and ethnicity categories

This supplementary material has been provided by the authors to give readers additional information about their work.

© 2024 Steel TL et al. *JAMA Network Open.*

eTable 1. ICD-10 Codes used to define alcohol withdrawal syndrome (AWS), chronic ambulatory care sensitive conditions (ACSCs), and common outpatient diagnoses associated with alcohol use disorder (AUD)

Study Measure	ICD-10 Codes
AWS	F1023x, F1013x, F1093x
Chronic ACSCs	
Heart failure	I0981, I110, I130, I132, I501, I5020, I5021, I5022, I5023, I5030, I5031, I5032, I5033, I5040, I5041, I5042, I5043, 1509, I50810, I50811, I50812, I50813, I50814, I5082, I5083, I5084, I5089
Chronic obstructive pulmonary disease	J410, J411, J418, J42, J430, J431, J432, J438, J439, J440, J441, J449, J470, J471, J479
Diabetes	E1010, E1101, E1011, E1110, E10641, E1111, E1100, E11641, E1021, E1121, E1022, E1122, E1029, E1129, E10311, E11311, E10319, E11319, E103211, E113211, E103211, E113211, E103212, E113212, E103213, E113213, E103219, E113219, E10329, E11329, E103291, E113291, E103292, E113292, E103293, E113293, E103299, E113299, E10331, E11331, E103311, E113311, E103312, E113312, E103313, E113313, E103319, E113319, E10339, E11339, E103391, E113391, E103392, E113392, E103393, E113393, E103399, E113399, E10341, E11341, E103411, E113411, E103412, E113412, E103413, E113413, E103419, E113419, E10349, E11349, E103491, E113491, E103492, E113492, E103493, E113493, E103499, E113499, E11351, E103511, E113511, E103512, E113512, E103513, E113513, E103519, E113519, E103521, E113521, E103522, E113522, E103523, E113523, E103529, E113529, E103531, E113531, E103532, E113532, E103533, E113533, E103539, E113539, E10354, E113541, E103542, E113542, E103543, E113543, E103549, E113549, E103551, E113551, E103552, E113552, E103553, E113553, E103559, E113559, E10359, E11359, E103591, E113591, E103592, E113592, E103593, E113593, E103599, E113599, E1036, E1136, E1037X1, E1137X1, E1037X2, E1137X2, E1037X3, E1137X3, E1037X9, E1137X9, E1039, E1139, E1040, E1140, E1041, E1141, E1042, E1142, E1043, E1143, E1044, E1144, E1049, E1149, E1051, E1151, E1052, E1152, E1059, E1159, E10610, E11610, E10618, E11618, E10620, E11620, E10621, E11621, E10622, E11622, E10628, E11628, E10630, E11630, E10638, E11638, E1069, E1169, E108, E118
Hypertension	I160, I161, I169
Outpatient diagnoses	
Alcohol-attributable diagnosis	F10.3, F10.4, F10.5, F10.6, F10.7, F10.8, F10.9, F10.920, F10.921, F10.929, F10.10, F10.11, F10.120, F10.121, F10.129, F10.14, F10.150, F10.151, F10.159, F10.180, F10.182, F10.188, F10.19, F10.20, F10.220, F10.221, F10.229, F10.230, F10.231, F10.232, F10.239, F10.24, F10.250, F10.251, F10.259, F10.26, F10.27, F10.280, F10.282, F10.288, F10.29, G62.1, G31.2, G72.1, I42.6, K29.20, K29.21, K70.0, K70.10, K70.11, K70.2, K70.30, K70.31, K70.40, K70.9, K85.20, K85.21, K85.22, K86.0
Alcohol use disorder	F10.10, F10.120, F10.121, F10.129, F10.14, F10.150, F10.151, F10.159, F10.180, F10.181, F10.182, F10.188, F10.19, F10.20, F10.220, F10.221, F10.229, F10.230, F10.231, F10.232, F10.239, F10.24, F10.250, F10.251, F10.259, F10.26, F10.27, F10.280, F10.281, F10.282, F10.288, F10.29
Anxiety disorder	F06.4, F40.00, F40.01, F40.02, F40.10, F40.11, F40.210, F40.218, F40.220, F40.228, F40.230, F40.231, F40.232, F40.233, F40.240, F40.241, F40.242, F40.243, F40.248, F40.290, F40.291, F40.298, F40.8, F40.9, F41.0, F41.1, F41.3, F41.8, F41.9, F42.2, F42.3, F42.4, F42.8, F42.9, F43.0, F43.20, F43.21, F43.22, F43.23, F43.24, F43.25, F43.29, F43.8, F43.9, F93.0
Cancer (any)	C0x.x, C1x.x, C2x.x, C30.x, C31.x, C32.x, C33.x, C34.x, C37.x, C38.x, C39.x, C40.x, C41.x, C43.x, C45.x, C46.x, C47.x, C48.x, C49.x, C50, C51-58.x, C60-63.x, C76.x, C80.1, C81.x, C82.x, C83.x, C84.x, C85.x, C88.x, C9x.x
Cerebrovascular disease	G46.x, H34.0x, H34.1x, H34.2x, I60.x, I61.x, I62.x, I63.x, I64.x, I65.x, I66.x, I67.x, I68.x
Chronic kidney disease	I12.0, I12.9, I13.0, I13.10, I13.11, I13.2, N03.x, N05.x, N18.1, N18.2, N18.3, N18.4, N18.5, N18.6, N18.9, N19.x, N25.0, Z49.x, Z94.0, Z99.2
Chronic liver disease	B18.x, I85.0x, I86.4, K70.0, K70.1, K70.2, K70.3, K70.4x, K70.9, K71.1x, K71.3, K71.4, K71.5, K71.7, K72.1x, K72.9x, K73.x, K74.x, K76.0, K76.2, K76.3, K76.4, K76.5, K76.6, K76.7, K76.8, K76.9, Z94.4
Chronic pulmonary conditions	J40.x, J41.x, J42.x, J43.x, J44.x, J45.x, J46.x, J47.x, J60.x, J61.x, J62.x, J63.x, J64.x, J65.x, J66.x, J67.x, J68.4, J70.1, J70.3
Congestive heart failure	I13.0, I13.2, I25.5, I42.0, I42.5, I42.6, I42.7, I42.8, I42.9, I43.x, I50.x, P29.0
Dementia	F01.x, F02.x, F03.x, F04, F05, F06.1, F06.8, G13.2, G13.8, G30.x, G31.0x, G31.1, G31.2, G91.4, G94, R41.81, R54
Diabetes	E08.x, E09.x, E10.x, E11.x, E13.x
Mood disorder	F06.30, F06.31, F06.32, F06.33, F06.34, F06.34, F30.10, F30.11, F30.12, F30.13, F30.2, F30.3, F30.8, F30.9, F31.0, F31.10, F31.11, F31.12, F31.13, F31.2, F31.30, F31.31, F31.32, F31.4, F31.5, F31.60, F31.61, F31.62, F31.63, F31.64, F31.71, F31.73, F31.75, F31.77, F31.81, F31.89, F31.9, F32.0, F32.1,

© 2024 Steel TL et al. *JAMA Network Open.*

	F32.2, F32.3, F32.4, F32.8, F32.8, F32.89, F32.9, F32.A, F33.0, F33.1, F33.2, F33.3, F33.41, F33.8, F33.9, F34.0, F34.1, F34.8, F34.8, F34.89, F34.9, F39, O90.6
Nicotine use disorder	F17.211, F17.221, F17.291
Other substance use disorder	F11.10, F11.120, F11.121, F11.122, F11.129, F11.14, F11.150, F11.151, F11.159, F11.181, F11.182, F11.188, F11.19, F11.20, F11.220, F11.221, F11.222, F11.229, F11.23, F11.24, F11.250, F11.251, F11.259, F11.281, F11.282, F11.288, F11.29, F11.90, F11.920, F11.921, F11.922, F11.929, F11.93, F11.94, F11.950, F11.951, F11.959, F11.981, F11.982, F11.988, F12.10, F12.120, F12.121, F12.122, F12.129, F12.150, F12.151, F12.159, F12.180, F12.188, F12.19, F12.20, F12.220, F12.221, F12.222, F12.229, F12.250, F12.251, F12.259, F12.280, F12.288, F12.29, F13.10, F13.120, F13.121, F13.129, F13.14, F13.150, F13.151, F13.159, F13.180, F13.181, F13.182, F13.188, F13.19, F13.20, F13.220, F13.221, F13.229, F13.230, F13.231, F13.232, F13.239, F13.24, F13.250, F13.251, F13.259, F13.26, F13.27, F13.280, F13.281, F13.282, F13.288, F13.29, F14.10, F14.120, F14.121,F14.122,F14.129,F14.14, F14.150,F14.151, F14.159, F14.180, F14.181, F14.182, F14.188, F14.19, F14.20,F14.220, F14.221, F14.222, F14.229, F14.23, F14.24, F14.250, F14.251, F14.259, F14.280, F14.281, F14.282, F14.288, F14.29, F15.10, F15.120, F15.121, F15.122, F15.129, F15.14, F15.150, F15.151, F15.159, F15.180, F15.181, F15.182, F15.188, F15.19, F15.20, F15.220, F15.221, F15.222, F15.229, F15.23, F15.24, F15.250,F15.251, F15.259, F15.280, F15.281, F15.282, F15.288, F15.29, F16.10, F16.120, F16.121, F16.122, F16.129,F16.14, F16.150, F16.151, F16.159, F16.180, F16.183, F16.188, F16.19, F16.20, F16.220, F16.221, F16.229, F16.24, F16.250, F16.251, F16.259, F16.280, F16.283, F16.288, F16.29, F18.10, F18.120, F18.121, F18.129, F18.14 , F18.150, F18.151, F18.159, F18.17, F18.180, F18.188, F18.19, F18.20, F18.220, F18.221, F18.229, F18.24, F18.250, F18.251, F18.259, F18.27, F18.280, F18.288, F18.29, F19.10, F19.120, F19.121, F19.122, F19.129, F19.14, F19.150, F19.151, F19.159, F19.16, F19.17, F19.180, F19.181, F19.182, F19.188, F19.19, F19.20, F19.220, F19.221, F19.222, F19.229 ,F19.230 ,F19.231, F19.232, F19.239, F19.24, F19.250 , F19.251, F19.259, F19.26, F19.27, F19.280, F19.281, F19.282, F19.288, F19.29
Peptic ulcer disease	K25.x, K26.x, K27.x, K28.x
Post-traumatic stress disorder	F43.10, F43.11, F43.12
Psychotic disorder	F06.0, F06.1, F06.2, F20.0, F20.1, F20.2, F20.3, F20.5, F20.81, F20.89, F20.9, F21, F22, F23, F24, F25.0, F25.1, F25.8, F25.9, F28, F29

© 2024 Steel TL et al. *JAMA Network Open.*

eTable 2. Incidence and Proportional Incidence of hospitalizations involving alcohol withdrawal syndrome (AWS), overall and by patient characteristics, including all-cause hospitalization data

	AWS hospitalizations per 100,000 person-enrolled years		All-cause hospitalizations per 100,000 person-enrolled years		PI of AWS hospitalizations	
	IR	(95% CI)	IR	(95% CI)	PI	(95% CI)
All	168.6	(158.5-179.2)	7306.5	(7238.6-7375.8)	2.3	(2.2-2.4)
Age						
18-29	88.9	(73.6-105.7)	4293.4	(4182.2-4408)	2.1	(1.7-2.5)
30-39	167.6	(143.4-193.2)	5304.9	(5184.3-5428.4)	3.2	(2.7-3.6)
40-49	229.5	(197.1-263.8)	3401.3	(3290.4-3515)	6.7	(5.8-7.7)
50-59	231.9	(203.7-262.5)	4975.1	(4829-5121.4)	4.7	(4.1-5.2)
60-69	178.6	(155.1-203.5)	8202.1	(8025-8374.3)	2.2	(1.9-2.5)
70+	104.0	(84.3-125.4)	19517.3	(19228.1-19807.5)	0.5	(0.4-0.6)
Sex						
Female	116.6	(104.6-129)	7696.1	(7606.1-7786.7)	1.5	(1.4-1.7)
Male	236.6	(218.3-254.9)	6797.7	(6697.4-6901.8)	3.5	(3.2-3.7)
Race						
American Indian or Alaska Native	218.4	(98.5-362.5)	9040.4	(8148.8-9966.3)	2.4	(1.1-3.9)
Asian	22.4	(12.2-35.3)	4504.7	(4355.8-4656.9)	0.5	(0.3-0.8)
Black	124.0	(88.2-164.8)	8085.2	(7663.8-8529.2)	1.5	(1.1-2.0)
Native Hawaiian or Pacific Islander	14.7	(0.0-36.9)	7436.2	(6716.1-8173.4)	0.2	(0.0-0.5)
White	196.9	(74.6-163.6)	7973.9	(5910.3-6606.8)	2.5	(1.2-2.6)
Multiracial	116.8	(183.3-210.6)	6253.6	(7890.4-8057.8)	1.9	(2.3-2.6)
Other	81.5	(51.7-115.4)	5434.5	(5126.1-5767.0)	1.5	(1.0-2.1)
Unknown	197.9	(159.8-239.4)	5342.2	(5133.6-5558.3)	3.7	(3.0-4.4)
Ethnicity						
Latinx	151.1	(113.0-193.1)	6220.3	(5961.2-6481.4)	2.4	(1.8-3.1)
Not Latinx	166.2	(154.7-177.7)	7765.7	(7686.1-7844.5)	2.1	(2.0-2.3)
Unknown	192.9	(163.5-225.4)	4790.4	(4648.3-4934.7)	4.0	(3.4-4.7)
Outpatient Diagnoses						
Alcohol-attributable diagnosis	15346.7	(13502.2-17331.4)	39043.7	(36024.1-42182.8)	39.3	(35.8-42.9)
Alcohol use disorder	5088.9	(4763.1-5419.8)	19952.7	(19247.0-20649.3)	25.5	(24.1-26.9)
Anxiety disorder	398.3	(367.9-429.8)	9789.7	(9635.5-9943.2)	4.1	(3.8-4.4)
Cancer (any)	188.0	(146.3-235.6)	20977.3	(20556.7-21414.3)	0.9	(0.7-1.1)
Cerebrovascular disease	421.4	(337.8-514.6)	33364.4	(32618.5-34108.9)	1.3	(1.0-1.5)
Chronic kidney disease	263.2	(208.4-325.3)	30873.6	(30281.1-31481.5)	0.9	(0.7-1.1)
Chronic liver disease	1226.9	(1102.8-1361.8)	19829.3	(19317.0-20360.9)	6.2	(5.6-6.8)
Chronic pulmonary conditions	282.5	(249.1-317.0)	14534.2	(14269.2-14809.0)	1.9	(1.7-2.2)
Dementia	536.4	(401.8-689.7)	42988.9	(41845.3-44192.4)	1.2	(0.9-1.6)
Diabetes	228.9	(192.7-267.9)	17937.5	(17588.5-18298.2)	1.3	(1.1-1.5)
Heart failure	525.3	(433.4-626.6)	48913.7	(48013.1-49840.2)	1.1	(0.9-1.3)
Mood disorder	508.5	(467.5-551.4)	12398.3	(12184.9-12614.4)	4.1	(3.8-4.4)
Nicotine use disorder	1051.3	(953.5-1155.3)	14615.9	(14192.5-15043.9)	7.2	(6.5-7.9)
Other substance use disorder	2385.1	(2101.4-2682.0)	26317.2	(25200.4-27505.2)	9.1	(8.0-10.1)
Peptic ulcer disease	937.3	(692.0-1200.9)	37045.8	(35057.3-39128.3)	2.5	(1.9-3.3)
Posttraumatic stress disorder	853.4	(702.7-1014.8)	12389.7	(11807.9-12995.7)	6.9	(5.7-8.1)
Psychotic disorder	1256.2	(914.9-1639.3)	33212.9	(31234.2-35236.7)	3.8	(2.8-4.9)
Alcohol consumption (outpatient AUDIT-C scores, 0-12 points)						
Very high-risk use (9-12 points)	5754.5	(5023.4-6543.8)	13219.1	(12041.0-14446.2)	43.5	(39.5-47.6)
High-risk use (7-8 points)	1241.7	(1036.6-1466.1)	5518.2	(5075.0-5971.3)	22.5	(19.2-26.0)
Unhealthy use (3-6 points female/4-6 points male)	210.3	(187.1-235.1)	5057.6	(4950.7-5167.9)	4.2	(3.7-4.6)
Low use (1-2 points female/1-3 points male)	59.0	(49.9-68.7)	6213.5	(6112.5-6313.3)	0.9	(0.8-1.1)
No use (0 points)	91.4	(75.7-108.8)	11213.9	(11039.3-11387.8)	0.8	(0.7-1.0)
No AUDIT-C screen completed	152.8	(127.2-180.0)	6684.6	(6491.1-6886.3)	2.3	(1.9-2.7)

Abbreviations: AUDIT-C=Alcohol Use Disorder Identification Test–Consumption, AWS=alcohol withdrawal syndrome, CI=confidence interval, IR=incidence rate, PI=proportional incidence

© 2024 Steel TL et al. *JAMA Network Open.*

eTable 3. Proportional Incidence of hospitalizations with alcohol withdrawal syndrome (AWS) relative to all-cause hospitalizations across age groups, by sex

	**Female**		**Male**	
	PI	(95% CI)	PI	(95% CI)
18-29	1.0	(0.7-1.3)	6.3	(5.0-7.7)
30-39	1.6	(1.3-2.0)	10.5	(8.8-12.3)
40-49	4.8	(3.7-5.8)	9.4	(7.9-11.1)
50-59	3.4	(2.7-4.3)	6.0	(5.2-6.9)
60-69	1.6	(1.3-2.0)	2.7	(2.3-3.2)
70+	0.3	(0.2-0.5)	0.8	(0.6-1.0)

Abbreviations: CI=confidence interval, PI=proportional incidence

© 2024 Steel TL et al. *JAMA Network Open.*

eTable 4. Proportional Incidence of hospitalizations with alcohol withdrawal syndrome (AWS) relative to all-cause hospitalizations across age groups, by race

	**AI/AN**		**Asian**		**Black**		**PI/NH**		**White**		**Multiracial**		**Other race**		**Unknown race**	
	PI	(95% CI)	PI	(95% CI)	PI	(95% CI)	PI	(95% CI)	PI	(95% CI)	PI	(95% CI)	PI	(95% CI)	PI	(95% CI)
18-29	5.3	(0.0-11.0)	0.1	(0.0-0.4)	1.7	(0.5-3.4)	0.0	(0.0-0.5)	2.3	(1.8-2.8)	1.7	(0.5-3.4)	1.1	(0.3-2.0)	3.5	(1.9-5.4)
30-39	3.0	(0.0-10.0)	0.6	(0.2-1.0)	1.2	(0.4-2.3)	0.0	(0.0-0.0)	3.9	(3.2-4.5)	1.6	(0.6-2.9)	1.5	(0.5-2.7)	5.5	(3.5-7.8)
40-49	4.5	(0.0-14.1)	0.3	(0.0-0.8)	3.3	(0.9-6.5)	1.1	(0.0-2.9)	8.0	(6.8-9.2)	8.5	(3.7-14.0)	4.4	(1.5-8.2)	8.3	(5.0-12.2)
50-59	3.8	(0.7-8.2)	2.4	(0.8-4.5)	2.6	(1.5-4.1)	0.0	(0.0-0.0)	5.3	(4.6-6.1)	2.1	(0.3-4.6)	0.8	(0.0-1.8)	5.2	(3.5-7.1)
60-69	1.1	(0.0-2.8)	0.2	(0.0-0.5)	1.0	(0.4-1.7)	0.0	(0.0-0.0)	2.4	(2.1-2.8)	0.7	(0.0-2.0)	1.6	(0.4-3.0)	2.2	(1.0-3.6)
70+	0.8	(0.0-2.1)	0.1	(0.0-0.2)	0.5	(0.1-1.2)	0.0	(0.0-0.0)	0.6	(0.5-0.7)	0.3	(0.0-0.9)	0.2	(0.0-0.8)	0.6	(0.1-1.4)

Abbreviations: AI/AN=American Indian/Alaska Native, CI=confidence interval, NH/PI=Native Hawaiian or Pacific Islander, PI=proportional incidence

eTable 5. Proportional Incidence of hospitalizations with alcohol withdrawal syndrome (AWS) relative to all-cause hospitalizations across age groups, by Alcohol Use Disorder Identification Test-Consumption (AUDIT-C) category

	**No Alcohol Use** AUDIT-C 0 points		**Low-risk Alcohol Use** AUDIT-C 1-2 points female/ 1-3 points male		**Unhealthy Alcohol Use** AUDIT-C 3-6 points female/ 4-6 points male		**High-risk Alcohol Use** AUDIT-C 7-8 points		**Very High-risk Alcohol Use** AUDIT-C 9-12 points	
	PI	(95% CI)	PI	(95% CI)	PI	(95% CI)	PI	(95% CI)	PI	(95% CI)
18-29	0.7	(0.4-1.1)	0.7	(0.3-1.2)	2.7	(1.8-3.6)	21.9	(12.4-32.5)	31.1	(22.5-40.9)
30-39	1.1	(0.7-1.4)	1.1	(0.7-1.5)	4.1	(2.9-5.3)	26.5	(17.5-35.9)	47.7	(38.7-56.3)
40-49	2.4	(1.5-3.5)	2.1	(1.3-3.0)	10.6	(7.9-13.4)	32.4	(22.4-42.5)	49.5	(40.6-58.8)
50-59	2.0	(1.3-3.0)	1.9	(1.3-2.5)	7.5	(5.9-9.2)	26.3	(19.8-33.0)	51.0	(43.4-58.6)
60-69	0.9	(0.6-1.2)	1.1	(0.8-1.4)	4.9	(3.8-6.1)	15.7	(10.4-21.4)	38.8	(30.2-47.7)
70+	0.2	(0.1-0.3)	0.3	(0.2-0.4)	1.6	(1.1-2.0)	9.5	(5.0-14.8)	26.7	(13.3-42.0)

Abbreviations: AUDIT-C=Alcohol Use Disorder Identification Test–Consumption, CI=confidence interval, PI=proportional incidence

© 2024 Steel TL et al. *JAMA Network Open.*

eTable 6. Proportional Incidence (PI) of Primary ICD-10 diagnosis codes only for alcohol withdrawal syndrome (AWS), heart failure, chronic obstructive pulmonary disease (COPD), diabetes, and hypertension during hospitalizations relative to all-cause hospitalizations across age, race, and ethnicity categories

	**AWS**		**COPD**		**Diabetes**		**Heart failure**		**Hypertension**	
	PI	(95% CI)	PI	(95% CI)	PI	(95% CI)	PI	(95% CI)	PI	(95% CI)
All	0.7%	(0.6-0.8%)	0.7%	(0.6-0.8%)	0.9%	(0.8-1.0%)	2.5%	(2.3-2.6%)	0.3%	(0.3-0.3%)
Age										
18-29	0.6%	(0.4-0.8%)	0.0%	(0.0-0.0%)	1.5%	(1.1-2.1%)	0.1%	(0.0-0.1%)	0.1%	(0.0-0.2%)
30-39	1.1%	(0.9-1.4%)	0.0%	(0.0-0.0%)	0.5%	(0.4-0.7%)	0.2%	(0.1-0.3%)	0.1%	(0.1-0.2%)
40-49	2.2%	(1.8-2.7%)	0.1%	(0.1-0.2%)	1.5%	(1.1-2.1%)	1.1%	(0.8-1.4%)	0.4%	(0.3-0.5%)
50-59	1.5%	(1.3-1.8%)	0.8%	(0.5-1.1%)	1.3%	(1.0-1.5%)	1.5%	(1.2-1.7%)	0.4%	(0.3-0.5%)
60-69	0.5%	(0.4-0.6%)	1.0%	(0.8-1.1%)	1.0%	(0.8-1.1%)	2.1%	(1.8-2.3%)	0.3%	(0.3-0.4%)
70+	0.1%	(0.1-0.2%)	1.0%	(0.9-1.2%)	0.5%	(0.4-0.6%)	4.6%	(4.4-4.9%)	0.3%	(0.3-0.4%)
Sex										
Female	0.5%	(0.4-0.5%)	0.6%	(0.6-0.7%)	0.6%	(0.5-0.7%)	2.0%	(1.9-2.2%)	0.3%	(0.2-0.3%)
Male	1.0%	(0.9-1.2%)	0.8%	(0.6-0.9%)	1.3%	(1.2-1.5%)	3.1%	(2.9-3.3%)	0.3%	(0.2-0.3%)
Race										
AI/AN	0.6%	(0.1-1.3%)	0.6%	(0.1-1.3%)	0.8%	(0.2-1.6%)	1.8%	(0.9-2.8%)	0.6%	(0.1-1.2%)
Asian	0.2%	(0.1-0.4%)	0.1%	(0.0-0.2%)	0.6%	(0.4-0.8%)	1.8%	(1.4-2.2%)	0.3%	(0.2-0.5%)
Black	0.3%	(0.1-0.5%)	0.6%	(0.4-1.0%)	1.4%	(0.8-2.4%)	3.0%	(2.4-3.5%)	0.7%	(0.5-0.9%)
NH/PI	0.0%	(0.0-0.0%)	0.7%	(0.2-1.3%)	1.9%	(1.0-2.8%)	2.5%	(1.3-3.8%)	1.0%	(0.4-1.7%)
Multiracial	0.7%	(0.6-0.8%)	0.8%	(0.7-0.8%)	0.8%	(0.7-0.9%)	2.6%	(2.4-2.7%)	0.2%	(0.2-0.3%)
White	0.6%	(0.3-0.9%)	0.7%	(0.3-1.2%)	1.3%	(0.8-1.9%)	2.1%	(1.5-2.8%)	0.3%	(0.1-0.5%)
Other	0.5%	(0.2-0.8%)	0.5%	(0.2-0.9%)	0.8%	(0.5-1.3%)	1.5%	(0.9-2.2%)	0.3%	(0.1-0.6%)
Unknown	1.5%	(1.1-2.0%)	0.4%	(0.2-0.6%)	1.3%	(1.0-1.7%)	1.8%	(1.4-2.2%)	0.2%	(0.1-0.4%)
Ethnicity										
Hispanic	0.9%	(0.6-1.2%)	0.3%	(0.2-0.5%)	0.9%	(0.6-1.2%)	1.6%	(1.2-1.9%)	0.2%	(0.1-0.3%)
Not Hispanic	0.6%	(0.5-0.7%)	0.7%	(0.7-0.8%)	0.8%	(0.8-0.9%)	2.6%	(2.5-2.7%)	0.3%	(0.2-0.3%)
Unknown	1.5%	(1.1-1.9%)	0.5%	(0.3-0.7%)	1.2%	(0.9-1.4%)	1.6%	(1.2-1.9%)	0.3%	(0.2-0.5%)

Abbreviations: AI/AN=American Indian/Alaska Native, AWS=alcohol withdrawal syndrome; CI=confidence interval, ICD-10=International Classification of Diseases-10th revision, NH/PI=Native Hawaiian or Pacific Islander, PI=proportional incidence

© 2024 Steel TL et al. *JAMA Network Open.*
