# Supplementary material for: Incidence of Hospitalizations Involving Alcohol Withdrawal Syndrome in a Primary Care Population
Source: JAMA Netw Open. 2024 Oct 8;7(10):e2438128. doi: 10.1001/jamanetworkopen.2024.38128 (PMC11581492; doi:10.1001/jamanetworkopen.2024.38128)
Supplement: Supplement 2. — Data Sharing Statement [file jamanetwopen-e2438128-s002.pdf]

Data Sharing Statement

Steel. Incidence of Hospitalizations Involving Alcohol Withdrawal Syndrome in a Primary Care Population. *JAMA Netw Open*. Published October 08, 2024.
doi:10.1001/jamanetworkopen.2024.38128

Data
Data available: No
